# Effect of Moxibustion on Behavioral Changes and Expression of APP and BACE1 in Hippocampus of SAMP8 Mice

**DOI:** 10.1155/2020/3598930

**Published:** 2020-08-14

**Authors:** Lue Ha, Bin Yang, Shaosong Wang, Yu An, Hao Wang, Yingxue Cui

**Affiliations:** ^1^School of Acupuncture, Moxibustion and Massage, Shaanxi University of Chinese Medicine, Xixian New Area, Xianyang 712046, China; ^2^Xiyuan Hospital, China Academy of Chinese Medical Sciences, Beijing 100091, China; ^3^Beijing TCM Hospital Affiliated to Capital Medical University, Beijing 100029, China; ^4^School of Acupuncture, Moxibustion and Tuina, Beijing University of Chinese Medicine, Beijing 100029, China

## Abstract

**Objective:**

To observe the effect of moxibustion on cognitive function of aging mice, to observe the effect of moxibustion on protein and gene expression of APP metabolism pathway, and to explore the mechanism of action in moxibustion.

**Methods:**

24 SAMP8 were randomly divided into 2 groups (12 in each group): moxibustion group and model group. 12 SAMR1 mice were used as blank controls. Mice in the moxibustion group were treated with moxibustion for 8 weeks, 10 minutes each time, 5 times a week, and for a total of 8 weeks. The model group and the blank group were treated with sham-moxibustion. Behavior tests were used to detect the learning and memory ability of each group of mice. Immunohistochemical, western blot, and RT-PCR were used to detect the protein and mRNA expression of APP and BACE1. Furthermore, the expressions of miR-29 and miR-101 were observed by RT-PCR method to explore the mechanism of moxibustion at the genetic level.

**Results:**

In this study, relative to normal mice, we found that aging mice showed behavioral changes consistent with the onset of AD. However, moxibustion interventions were able to mitigate these effects to some degree in aging mice. In addition, moxibustion was proved to regulate APP metabolism pathway at protein and gene level through molecular biology tests.

**Conclusion:**

The data suggest that the effect of moxibustion intervention on cognitive function in aging mice is related to the regulation of genes and proteins involved in APP metabolism pathway; this may be a potential target for treating Alzheimer's disease.

## 1. Introduction

Alzheimer's disease (AD) is the most prevalent neurodegenerative disorder worldwide. It is a multifactorial complex disease that cannot currently be treated, prevented, or slowed. It is characterized by A*β* deposition and the formation of intracellular neurofibrillary tangles (NFTs) in the brain regions involved in learning and memory [1]. In addition to the damage of patient's physical health, it also impairs patients' personal dignity and brings a heavy medical burden to their families [2]. At present, there is no specific drug for the treatment of AD, and conventional drugs generally only alleviate symptoms, while inevitably producing unwanted side effects [3]. Given these limitations, complementary and alternative therapies are attracting increasing attentions, among which the role of moxibustion in the prevention and treatment of AD has been recognized.

For thousands of years, moxibustion has been widely used in clinical and family health care in China and other Asian countries. It is a form of heat therapy and works through direct or indirect thermal stimulation over specific acupoints [4]. Recently, moxibustion has been used for treatment of several senile diseases [5, 6]; however few researches focus on the underlying mechanism.

Advancing age is one of the significant risk factors for the development of AD [7]. The senescence accelerated prone mouse (SAMP8) displays features of cognitive decline and Alzheimer's disease. It is a mouse strain that is used to study the effects of age on AD development [8] and has gained considerable attention in dementia research due to accelerated aging and exhibited AD phenotype [9].

Our previous studies have shown that moxibustion and moxa smoke can improve the antioxidant ability of SAMP8 mice [10–12] and regulate the abnormal metabolism of monoamine and amino acid neurotransmitters in the brain of SAMP8 mice [13]. In addition, moxibustion has also been found to delay the aging of SAMP8 mice by regulating MAPK pathway to improve the function of brain nerve cells [14]. Moreover, other studies have confirmed that moxibustion can enhance the learning and memory ability of SAMP8 mice [15].

Deposition of A*β* amyloid protein is a key mechanism for the development of cognitive impairment, and its main pathway is the hydrolysis of amyloid precursor protein APP into BACE1 (*β*-secretase 1). Therefore, regulating the amyloid production pathway plays an important role in inhibiting the occurrence and development of cognitive impairment [15].

MicroRNA (microRNA, or miRNA for short) is a series of noncoding small RNA with an endogenous length of 20–23 nucleotides, which negatively regulates mRNA expression after transcription [16, 17]. Research linking miRNA to pathological processes has exploded in recent years, and evidence is mounting that they have a role in neurodegenerative diseases ranging from AD to Parkinson's disease [18]. It was reported that electroacupuncture could reduce the protein expressions of APP and BACE1 in APP/PS1 double transgenic mice; however the mechanism had not been explored in this study [19]. It prompts us to think of whether moxibustion can produce a similar effect on APP and BACE1 production. And what is its potential mechanism? Evidence has shown that some microRNAs may inhibit amyloid production at the genetic level, among which miR-29 and miR-101 were negatively correlated with the protein expression of APP and BACE1 [20, 21], providing a new direction for revealing the mechanism of moxibustion in the treatment of AD.

Therefore, in this study, the rapidly aging SAMP8 mice were used as the AD model and moxibustion was used as intervention. Behavioral changes of AD mice were observed, and expression of APP and BACE1 was detected to explore the mechanism of moxibustion regulating AD from the amyloid production pathway. Moreover, by observing the expression of miR-29 and miR-101, we can further verify the role of moxibustion at the genetic level in amyloid production pathway.

## 2. Materials and Methods

### 2.1. Animals

24 male 6-week-old SAMP8 mice were selected as the AD animal model, and 12 of the same genetic background SAMR1 mice were used as a negative control. All mice (SCXK (Jing) 2014–0011) were six months of age and were provided by Beijing ZhongKe ZeSheng Technology Co., Ltd., China. Mice were housed in separate cages under normal controlled conditions (i.e., temperature 22 ± 2°C, humidity 55%, and a 13-hour daily photoperiod from 6 : 00 to 19 : 00) and were given free access to food pellets and water.

Ethical approval of this study was obtained from the Medicine and Animal Ethics Committee in Beijing University of Chinese Medicine. All experiments were performed according to the National Guideline for the Care and Use of Laboratory Animals, Amendment 2 (State Council of China, 2013).

### 2.2. Experiment Design

**Figure 1:**
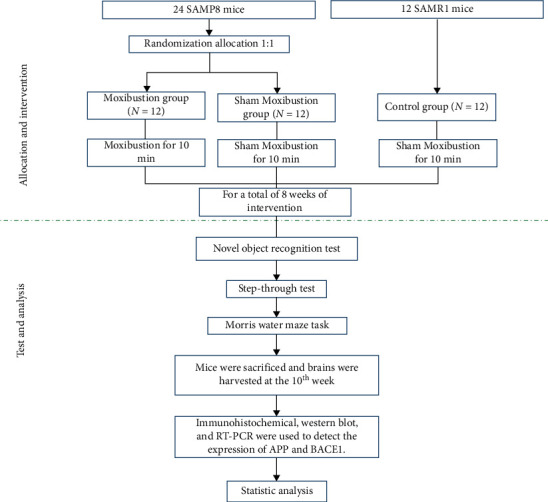
Flow diagram of study design.

After one-week adaptive feeding, 24 SAMP8 mice were randomly assigned (i.e., by a random number table) to two groups (*n* = 12 per group): moxibustion (M) and sham-moxibustion (SM). In addition, 12 SAMR1 mice served as a normal (negative) control group (C). Mice in the moxibustion group received moxibustion at Guanyuan (CV 4) acupoint, 10 min per day, 5 days per week, and for a total of 8 weeks. Behavioral tests were implemented in the 9^th^ week, 3 days after the completion of the moxibustion intervention. After the behavioral tests, the mice were sacrificed by cervical dislocation following deep anesthetization with 1% pentobarbital sodium (50 mg/kg body weight). Then, the brains were collected for immunochemistry staining, western blot, and real-time PCR testing. The experimental flow chart is shown in Figure 1.

### 2.3. Moxibustion Treatment

**Figure 2:**
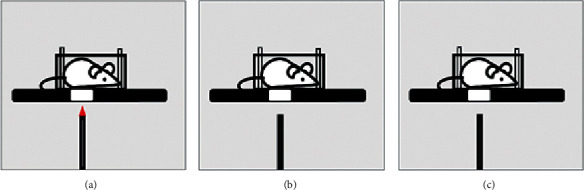
Intervention measures. (a) M. (b) SM. (c) C.

Mice in the moxibustion group received moxibustion with moxa stick under CV 4 (Guanyuan) for 10 minutes while being restrained in a plastic tube-shape holder, with the central part of the abdomen and the four limbs exposed. The moxa stick (*φ*0.5 cm × 20 cm, Henan Nanyang Hanyi Moxa Co., Ltd.) was ignited 2–3 cm under CV 4, which is located on the midline of the lower abdomen, 10 mm inferior to the umbilicus according to a standard atlas of mice acupuncture points [22]. The height of the moxa stick was adjusted continuously to maintain a constant distance so that the temperature could remain stable. Mice in the sham-moxibustion and control group were restrained in the holder with an unlit moxa stick under CV 4. The schematic diagram of moxibustion intervention is shown in Figure 2.

### 2.4. Behavioral Testing

#### 2.4.1. Novel Object Recognition Task

A white opaque uncovered glass box (50 cm × 50 cm×25 cm) was used as the experimental apparatus, and the roof was equipped with illumination and camera to record the mouse activity. Two wooden cubes (old objects) with identical shape and size were placed in the middle of the glass box at 10 cm away from the side wall of the glass box, respectively [23].

The experimental process was divided into adaptation period and test period; in the adaptation period, the mice were put into the glass box from the middle of the two test objects, allowed to explore for 5 min freely, and returned to the rearing cage. 24 h later, the test period was initiated, and one of the wooden cubes was replaced by a wooden cylinder (novel object) with identical size but different shape. Then, mice were put into the glass box at the same position for an exploration of 5 min, and it was deemed as the probe behavior when the mouse nose was <1 cm away from the object. The number and time of mice in exploring the old object and novel object during the test period were observed and the recognition index (number of novel object explorations/number of total explorations × 100%) and discrimination index (time of novel object explorations/time of total explorations × 100%) were recorded as results. After testing each animal, 75% ethanol was used to clean the glass box, so as to avoid the influence of the previous animal on the test results of the next one.

#### 2.4.2. Step-Through Test

The experimental apparatus of step-through test was constituted by a cuboid box, pedal and power device. The cuboid box was divided into light and dark rooms in the left and right sides, respectively, which were connected through a dodge gate, and the box bottom was the electric metal gate.

The experiment was divided into learning period and test period: day 1 was deemed as the learning period, in which mice were put into the experimental box to adapt for 3 min and allowed to move freely, and the box was not energized during this period. Subsequently, the mice were taken out, the dark box was energized (0.5 mA), and the mice were put from the light room with their backs to the dodge gate. If mice entered the dark room, they would be subjected to electric shock; the electric shock frequency (error number) of the mice within 5 min and the first time to enter the dark room (latency) were recorded. If mice did not enter the dark room within 5 min, the error number was recorded as 0, and the latency was 5 min. Day 2 of the experiment was the test period; the error number and latency of mice were recorded as described above.

#### 2.4.3. Morris Water Maze Test

Spatial learning (acquisition) and memory (retention) of mice were measured by a circle pool (diameter of 140 cm and height of 50 cm) filled with opaque water mixed with black food coloring with constant temperature (23°C–27°C) [24]. The maze is surrounded on all sides by curtains to avoid visual interference during trial. The acquisition trial consisted of 4 trials per day for 4 successive days and followed by one additional day for the probe trial. The goal quadrant contained a clear, 8 cm diameter submerge platform, 1 cm below the water. For the acquisition phase, mice were put into the water facing the side of the pool in 4 quadrants, respectively. Each trial was for a maximum of 60 s. Mice was allowed a 5 s stay on the platform if the animal found the platform; if not, it would be gently led to the platform by the experimenter and given a 30 s stay. The latency to find the platform is measured. For the probe trial, the platform was removed and animal was started at a novel position and had 60 s to investigate the maze. The dependent measure for the probe trial is the swimming distance in the target quadrant and times of crossing the platform.

### 2.5. Histological Staining

Brain samples were fixed in 10% buffered formalin at 4°C for 24–48 h and sectioned (6 *μ*m thickness) using a microtome (Leica RM2235) after paraffin embedding.

Standard immunohistochemical procedures were used to visualize APP and BACE1 expression. Sections were incubated overnight at 4°C with a primary antibody directed against APP (Bioworld, BS6418, 1 : 100) and BACE1 (Proteintech 12807-1-AP, 1 : 100) and then incubated for 30 minutes with goat anti-rabbit secondary antibody (Dako REAL EnVision). After DAB reaction for 3 min, the sections were counterstained with haematoxylin and eosin and viewed under a microscope (Olympus BX51). Images were captured (Nikon DS-U3) and quantified by two independent investigators blinded to experimental conditions. Image Pro Plus 6.0 software was used to obtain the mean optical density (MOD) which is widely used in the image analysis.

### 2.6. Western Blot Analysis

The extracted proteins were separated by electrophoresis with the 10% SDS-PAGE. Gel was run at 80V for 20 min and 120V for 60 min until samples run off the gel and then transferred onto PVDF membranes at 4°C at 80V for 1.5 h. The target protein APP and BACE-1 were measured using the primary antibody of anti-APP (Bioworld, BS6418, 1 : 100) and BACE1 (Proteintech 12807-1-AP, 1 : 100) and then incubated at 4°C overnight. After being washed three times with TBST, corresponding secondary antibody was used at a dilution of 1 : 2000 (bs-40295G, bs40296 G, Bioss, China), followed by visualization with ECL kit (mixed with 1 : 1, PE0010, Solarbio, China). The exposure was completed in dark room with chemiluminescence gel imaging system (C600, Azure Biosystems, USA). The antibody against GAPDH (1 : 2000, TA-08, Zsbio, China) and *β*-actin (1 : 2000, bs-0061R, Bioss, China) were used as internal controls. Quantitative results were expressed as a ratio of APP to *β*-actin and ratio of BACE-1 to GAPDH and then compared in each group to measure relative changes.

### 2.7. Real-Time PCR

Total RNA was extracted using mirVana™ RNA Isolation Kit (Applied Biosystems) according to the instructions of the manufacturer. The yield, purity, and quality of RNA were determined spectrophotometrically (NanoDrop, USA) and using the Bioanalyzer 2100 capillary electrophoresis. RNA samples from 16 individuals (four from each group: sedentary SAMR1, runner SAMR1, sedentary SAMP8, and runner SAMP8) were converted into cDNA through a reverse transcription reaction using miScriptIIRT Kit (Qiagen, Hilden Germany) according to the manufacturer's instructions. The expression of miRNAs was then analyzed using the miScript®miRNA PCR Array-Neurological Development and Disease miRNA PCR Array (Qiagen). miRNAs expression was measured in an ABI Prism7900HT through SYBR-green-real-time PCR.

### 2.8. Statistical Analysis

Data are expressed as means ± standard error. Groups were compared by one-way analysis of variance (ANOVA) followed by post hoc test of least significant difference using SPSS V.17.0 software. Multivariate analyses of variance are used to make comparisons between groups at each time point (with LSD post hoc tests used for pairwise comparisons). A probability level of *p* < 0.05 was set as the threshold of statistical significance.

## 3. Results

### 3.1. Behavioral Test

#### 3.1.1. Novel Object Recognition Task

**Figure 3:**
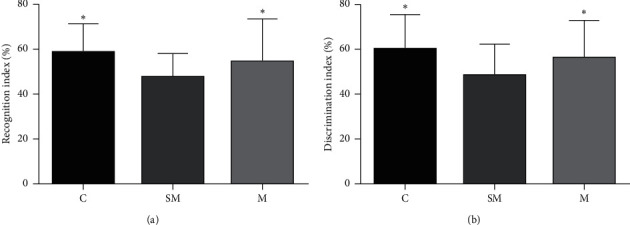
(a) Mean recognition index of each group. (b) Mean discrimination index of each group. Data are expressed as means ± SD (*n* = 12), ^*∗*^*p* < 0.05 versus SM group.

Our results showed that the recognition index of M group was significantly increased (*p* < 0.05) relative to the SM group (Figure 3(a)). In addition, the discrimination index was significantly increased (*p* < 0.05) compared to the SM group (Figure 3(b)). Compared to the SM group, recognition index and discrimination index of the C group were significantly increased (Figure 3(a)). There is no significant difference between the C group and M group (*p* > 0.05) (Figure 3(b)).

#### 3.1.2. Step-Through Test

**Figure 4:**
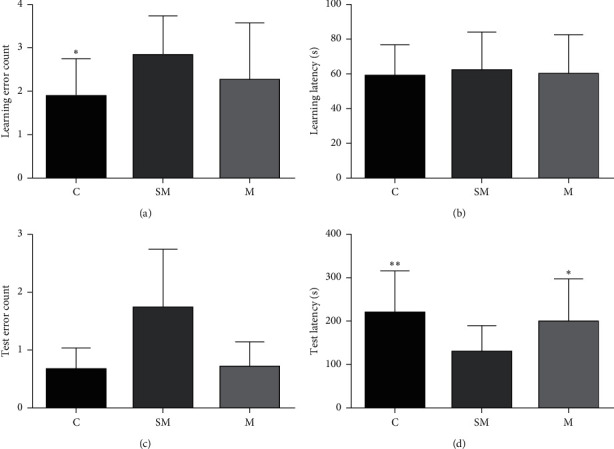
Step-through test results. (a) Mean number of errors of each group in the learning session. (b) Mean learning latency of each group in the learning session. (c) Mean number of errors of each group in the test session. (d) Mean test latency of each group in the test session. Data are expressed as means ± SD (*n* = 12). ^*∗*^*p* < 0.05 versus SM group, ^*∗∗*^*p* < 0.01 versus SM group.

In the learning session, the number of electric shocks (error count) was significantly higher in SM group compared to the C group (*p* < 0.05), while there was no significant difference between the C group and M group (*p* > 0.05) (Figure 4(a)). There was no statistical difference in the meantime of mice entering the dark room for the first time (latency) of all groups (*p* > 0.05) (Figure 4(b)).

In the test session, there was no statistical difference in the number of errors between the groups (Figure 4(c)). Compared to the SM group, the latency of mice in the M group and C group was significantly longer (*p* < 0.05, *p* > 0.05) (Figure 4(d)).

#### 3.1.3. Morris Water Maze Test

**Figure 5:**
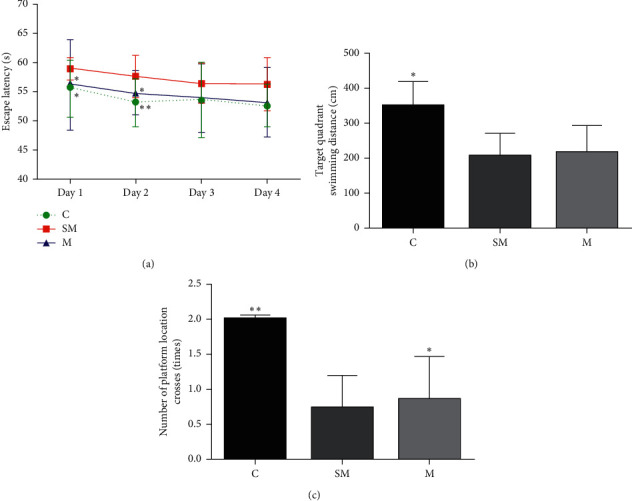
(a) Mean escape latency of each group for spatial learning test. (b) Mean target quadrant swimming distance of each group for the probe trial test. (c) Mean number of platform location crosses of each group for the probe trial test. Data are expressed as means ± SD (*n* = 12). ^*∗*^*p* < 0.05 versus SM group.

In the spatial learning test (day 1 to day 4), with the increase of training days, all mice had shorter escape latency. On day 1 and day 2, the latency of the mice in SM group was significantly longer than that in the C group (*p* < 0.05, *p* < 0.01), and compared with SM group, the latency of the mice in M group was significantly shorter (*p* < 0.05). There was no significant difference among the three groups on day 3 and day 4 (Figure 5(a)).

In the probe trial on day 5, the distance of mice swimming in the platform quadrant in SM group was significantly shorter than that in the C group (*p* < 0.05), and the distance of M group was slightly longer than that in SM group, with no significant difference (Figure 5(b)). The number of mice crossing the platform in SM group was significantly lower than that in the C group (*p* < 0.01) and M group (*p* < 0.05); there is no statistic difference between the number of mice in C and M group (Figure 5(c)).

### 3.2. The Expressions of APP and BACE1 in Each Group

**Figure 6:**
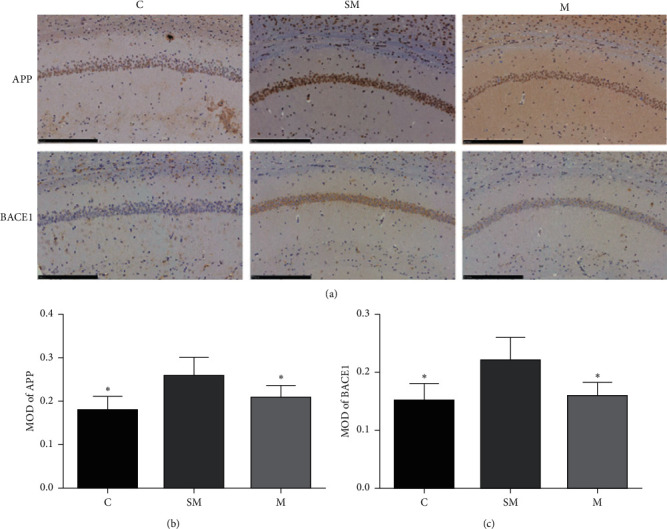
(a) Representative micrographs of immunohistochemical staining for APP and BACE1. (b) The result of image analysis of APP. (c) The result of image analysis of BACE1, MOD, and mean optical density. Data are expressed as means ± SD (*n* = 12). ^*∗*^*p* < 0.05 versus SM group.

Immunohistochemistry was used to detect the expression of APP and BACE1 protein in mice of each group. The results showed that compared with the SM group, APP content in the C group and M group decreased significantly (*p* < 0.05), Figure 6(b). Consistent with the results of APP, BACE1 content in the C group and M group decreased significantly (*p* < 0.05), Figure 6(c).

**Figure 7:**
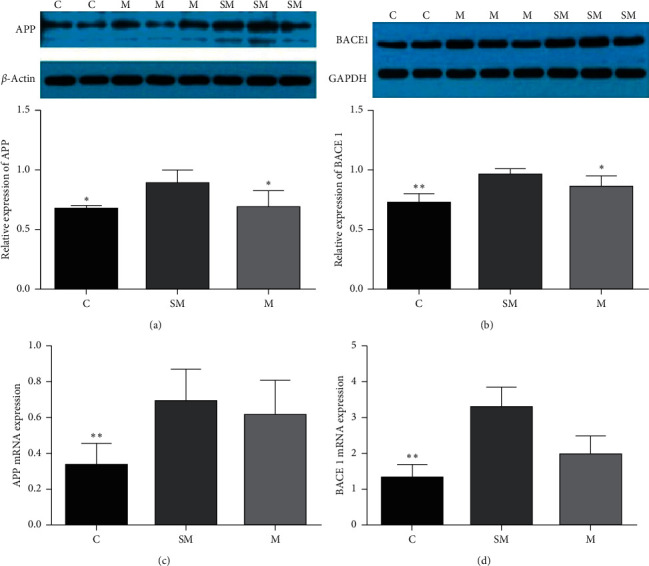
(a) The protein level of APP in each group. (b) The protein level of BACE1 in each group. (c) The mNRA expression of APP in each group. (d) The mNRA expression of BACE1 in each group. Data are expressed as means ± SD (*n* = 12). ^*∗*^*p* < 0.05 versus SM group, ^*∗∗*^*p* < 0.01 versus SM group.

Western blot method was used to detect the expressions of APP and BACE1 protein in the hippocampus of mice in each group. Compared with the C group, the hippocampal APP expression was significantly increased in SM group (*p* < 0.05). Compared with the SM group, the hippocampal APP expression in M group was significantly lower (*p* < 0.05). Compared with the C group, the hippocampal BACE1 expression was significantly increased in SM group (*p* < 0.01). Compared with the SM group, the hippocampal BACE1 expression in M group was significantly lower (*p* < 0.05). The expression trend of APP and BACE1 at mRNA level in each group was consistent with protein level (Figure 7).

### 3.3. The Expressions of miR-29 and miR-101 in Each Group

**Figure 8:**
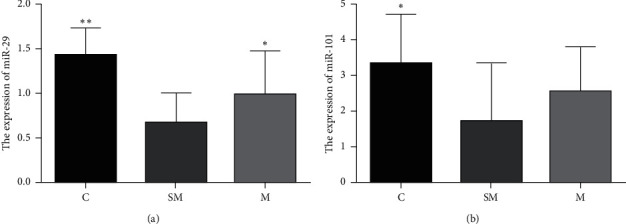
The expression of miR-29 (a) and miR-101 (b) in each group. Data are expressed as means ± SD (*n* = 12). ^*∗*^*p* < 0.05 versus SM group, ^*∗∗*^*p* < 0.01 versus SM group.

RT-PCR was used to detect the mRNA levels of miR-29 and miR-101 in each group. The results showed that the miR-29 expressions in C group were significantly higher than those in the SM group (*p* < 0.01), and the miR-29 expressions in M group were significantly higher relative to SM group (*p* < 0.05). The miR-101 expressions in C group were significantly higher than those in the SM group (*p* < 0.05), and the miR-101 expressions of M group were higher relative to SM group but showed no significant difference (Figure 8).

## 4. Discussion

In this study, general conditions of mice were continuously observed. All the mice were raised in separate cage, so there was abundance of room for activity. All the mice were in good mental status and acted freely with quick reaction, and no scratch marks were observed.

The average life span of SAMP8 is 10–12 months. Studies have shown that learning and memory ability of SAMP8 mice began to decrease from the age of 6 months after birth and showed a significantly decline at the age of 8 months [25]. Kanno et al. [26] reported the decline of spatial learning and memory by adopting water maze experiment started during the processes of 5 to 6 months of age of SAMP8 mice. Meanwhile, most studies have indicated that at the age of 8 months, SAMP8 mice present significant deficits in Morris water maze tasks compared with SAMR1 mice of the same age [27, 28]. The results showed that the characteristic feature of mild cognitive impairment could be measured as early as 6 months of age and SAMP8 mice develop severe deficits in learning and memory at the age of 8 months.

In this study, three behavioral tests were adopted to evaluate the effects of moxibustion on learning and memory in SAMP8 mice. The results in novel object recognition task (which has reflected the working memory) was consistent with Howlett's work [29]. Besides, we have found that moxibustion could extended test latency compared to M group in step-through test, illustrating that the moxibustion could enhance the passive avoidance reflex of the mice. In water maze experiment, our results are similar to Wang's research who had treated the streptozotocin-induced AD rat model with moxibustion at CV 4 acupoint, verifying that moxibustion at CV 4 acupoint could partially suppress the learning memory injury in the model rats [30]. The results of behavioral tests were consistent with our previous research [22] and they further prove that moxibustion can improve the cognitive dysfunction of AD mice.

According to traditional Chinese medicine, dementia is closely related to deficiency of Qi and blood in viscera and deficiency of kidney essence. CV 4 acupoint is one of the most important acupoints in human body, which has the effect of tonifying Qi and blood, invigorating kidney and brain. Wang [30] reported that moxibustion on CV 4 could partly improve learning and memory in AD rats, possibly through inhibition of hippocampal GSK-3*β* activities and downregulating the phosphorylation levels of Tau protein site Ser396/Thr231. Another study suggested that electroacupuncture at CV 4 could improve spatial learning and memory ability in female SAMP8 mice through regulating HPO axis [31].

It should also be noted that the intervention time point in this study is at the age of 6 months of mice which begin to develop mild cognitive impairment. Thus, this may be answer to the characteristics that moxibustion therapy is good at prevention and early intervention to improve the disease.

Abnormal protein aggregation in brain tissue is one of the mechanisms leading to the occurrence of cognitive disorder [32]. Pathologically, cognitive disorder can be accompanying with specific neuropathological changes, which manifest as massive accumulation and deposition of soluble *β*-amyloid (A*β*) in the brain, formation of senile plaques (SP), increased paired helical filaments (pHFs), and formation of neurofibrillary tangles (NFTs). The theory of A*β* cascade [33] holds that the overdeposition of A*β* in the brain is the initiating factor and central link of the occurrence and development of AD. A*β* deposition can cause brain parenchyma damage; the most significant one is hippocampus and other areas related to intelligence, thus causing learning and memory disorders [34]. Injection of A*β* 1–42 into the hippocampus can cause extensive neuronal degeneration in the CA3 area, which is manifested by neuronal loss and nuclear contraction in CA3, leading to impairment of learning and memory in the brain of AD mice [35]. Therefore, the amyloid protein production pathway (APP-BACE1-A*β*) is recognized as the key mechanism of the genesis of cognitive disorder, and clinging any link of this pathway is of vital importance to suppress the genesis and development of cognitive disorder.

The results of this study showed that the expressions of APP and BACE1 in M group were significantly lower than those in SM group, suggesting moxibustion therapy can reduce the formation and deposition of A*β* by inhibiting the amyloid production pathway.

In 2007, Walter J. Lukiw revealed the first clue into miRNA changes in AD, which provided a new entry point into the molecular basis of genetic forms of AD and illuminated the path toward novel therapeutic avenues [36]. The latest data showed specific changes in a series of miRNA in the AD brain, including miR-29, mir-181, mir-146, mir-9, mir-101, and mir-106, and these genes have been independently verified by two or more studies [37].

The miR-29 gene family is comprised of three categories, namely, miR-29a, miR-29b, and miR-29c. It has been currently verified in studies that the cognitive disorder-affected brain is accompanied with the downregulated miR-29 expression [38, 39]. In mouse brain, miR-29 is expressed at about 2 weeks after birth, which is gradually upregulated with age [40, 41]. BACE1 is a type of target gene of miR-29, which can bind with the 3'UTR of BACE1. Research suggests that low miR-29 expression can lead to reduction in the endogenous BACE1 protein level and increase of the subsequently produced A*β* deposit, while the BACE1 protein level is markedly reduced in transgene mice with overexpressed miR-29c [42]. Multiple target genes of miR-101 are closely involved in cognitive dysfunction. MiR-101 can specifically regulate the APP mRNA to directly suppress the APP production [43, 44]. It is found in some researches that miR-101 expression is reduced in the cognitive disorder-affected brain cortex, which leads to the increased APP expression and elevated production of A*β* lesion [21].

In this study, compared with control group, the expressions of miR-29 and miR-101 in SM group were significantly decreased, leading to the increase at APP and BECA1 protein level. These results are consistent with the negative regulation effect of miR-29 and miR101 on APP and BECA1 production [39, 43].

We acknowledge some deficiencies in this study. Our primary purpose of behavioral testing is to evaluate learning and memory ability; however, the stress/discomfort conditions of mice should also need behavioral evaluation, and sugar consumption experiments and open-field test should be conducted in the future research.

## 5. Conclusions

A mouse model of Alzheimer disease was established successfully in this study, and moxibustion intervention could significantly improve cognitive behaviors of AD mice. Amyloid production pathway plays an important role in the occurrence and development of cognitive impairment; it also might be associated with the therapeutic mechanism of moxibustion. This study has demonstrated that moxibustion could improve the learning and memory function of AD animals by regulating amyloid production pathway, which would be an effective regulation point for the efficacy of moxibustion.

## Data Availability

The data used to support the findings of this study are available from the corresponding author upon request.
